# Radial head fractures with interposed capitellar cartilage fragment–hindrance to bone healing–a case series

**DOI:** 10.1007/s00402-021-03895-z

**Published:** 2021-04-20

**Authors:** Andreas Harbrecht, Michael Hackl, Tim Leschinger, Kilian Wegmann, Dominik Seybold, Lars P. Müller

**Affiliations:** 1 Faculty of Medicine and University Hospital, Center for Orthopedic and Trauma Surgery, University of Cologne, Kerpener Str. 62, 50937 Cologne, Germany; 2 Department of General and Trauma Surgery, BG University Hospital Bergmannsheil, Bürkle-de-la-Camp Platz 1, 44789 Bochum, Germany

**Keywords:** Radial head fractures, Interposed capitellar cartilage, Osteosynthesis, Open reduction and internal fixation, Arthroscopy, Elbow surgery

## Abstract

**Introduction:**

Radial head fractures account for the majority of bony elbow injuries. The individual treatment options have been described in detail. In some cases, however, an unusual concomitant injury occurs, which can significantly impede primary osteosynthesis and healing. This concomitant injury can be an interposing cartilaginous capitellar fragment.

**Methods:**

This retrospective study describes four cases of trapped cartilage fragments of the capitellum that compromised primary osteosynthesis or primary conservative healing of a radial head fracture. Radiological imaging, function and pain level are presented pre- and postoperatively (mean follow-up 9.25 months).

**Results:**

None of the four cases showed preoperative evidence of an incarcerated cartilage fragment of the capitellum. They all showed limited elbow range of motion. CT examinations were performed in all cases. In each case, the cartilage fragment was first sighted upon surgery, subsequently removed and the fractures treated with ORIF. Mean follow-up was of 9.25 months. All fractures healed, with excellent function and low pain scores.

**Conclusions:**

This study presents rare cases of a trapped humeral cartilage fragment in radial head fractures. Radiological imaging including CT scans cannot reliably detect this concomitant injury. Therefore, this problem becomes apparent and treatable only during surgery. A high degree of suspicion is necessary especially in patients with minimally displaced fractures associated with limited elbow motion and a gap at the fracture site as treating these injuries conservatively may lead to poor outcome.

## Introduction

Radial head fractures have an overall incidence of 55.4 per 100,000 population and account for 5% of all fractures and one-third of all elbow fractures [1, 2]. The mechanism of injury is often a fall on the outstretched arm with the elbow partially flexed and forearm pronated [1]. The radial head then crushes into the capitellum, possibly fractures the distal humerus, leaves it unharmed or merely injures the cartilage [3–7]. One possible explanation for a cartilaginous shear fragment might be some degree of radiocapitellar subluxation. A cartilaginous shear injury is not visualized radiographically and may only be recognized at the time of surgery. Cartilage defects of the capitellum can be overlooked [8]. Incidence of cartilaginous defects vary from 19–29% [9, 10]. Loss of cartilage, however, can subsequently lead to arthrosis in the radiocapitellar joint as repetitive impact of the radial head on the defective capitellar articular surface occurs upon axial loading [11].

Radial head fractures can be classified by the modified Mason classification, which differentiates four types of fracture patterns [12].

Associated lesions, such as ligamentous injuries to the medial or lateral ligaments, elbow dislocations and additional fractures occur in 26% of radial head fractures [13]. Aim of any operative treatment must be the restoration of joint stability and function [14].

In this paper, we introduce four cases, three of them with interposing capitellar cartilage fragments blocking reduction and fixation of radial head fractures and one case of elbow stiffness after non-operative treatment of a radial head fracture, due to an interfering capitellar cartilage fragment.

## Methods

Three cases are presented, in which a capitellar cartilage shear fragment interfered with the reduction of a radial head fracture. These cases had seemingly isolated displaced radial head fractures that required fracture fixation. All cases were treated by the senior author (L.P.M.). One case was introduced to the hospital of Co-Author D.S. due to elbow stiffness after non-operative treatment of a radial head fracture. In the subsequent arthroscopic arthrolysis an interposing capitellar fragment could be detected (treatment by D.S.).

All cases were collected over a period of 5 years (2014–2019) and analyzed retrospectively. Radiographic material was evaluated to determine whether a capitellar shear fragment could have been detected before surgery. Fractures were categorized according to the modified Mason classification and intra-operative findings were documented [5, 12]. Concomitant ligamentous injuries were assessed by intra-operative inspection and/or stress tests under fluoroscopic imaging. The mean follow-up of patients was 9.25 months with a range of 2 to 26 months and consisted of function (range of motion) and pain evaluation (visual analogue scale for pain 0–10) as well as fracture healing.

An institutional ethics approval was granted prior to retrospective data collection (VT 20–1667). The study was performed in accordance with the ethical standards as laid down in the 1964 Declaration of Helsinki and its later amendments or comparable ethical standards.

## Results

Mean age of patients was 49 years (range 34–69 years). Two patients were female, two male (Table 1).

**Table 1:** Patient data and clinical outcome of radial head fractures with an interposed capitellar cartilage fragment

Case	Age (in y)	Sex	Side	Classification	Treatment	FU (in mo)	RoM	VAS	Compl	Revision surgery
#1	44	F	R	III	ORIF + LCLC	6	130°	0	–	None
#2	49	M	R	III	ORIF + LCLC	26	140°	0	–	None
#3	69	M	L	II	ASC + ORIF + LCLC	2	120°	0	–	None
#4	34	F	L	III	ASC + ORIF + LCLC	3	105°	2	–	None
*Mean*	*49*					*9.25*	*124°*	*0.5*		

*y * years, *M* male, *F* female, *R*  right, *L*  left, Classification according to modified Mason, *FU*  follow-up, *mo*  months, *RoM*  range of motion, *VAS*  visual analogue scale, *Compl.*  complications, *ASC * arthroscopy, *ORIF*  open reduction internal fixation via cannulated compression screw (CCS), *LCLC*  lateral collateral ligament complex reattached via anchors

### Case 1

**Fig. 1:**
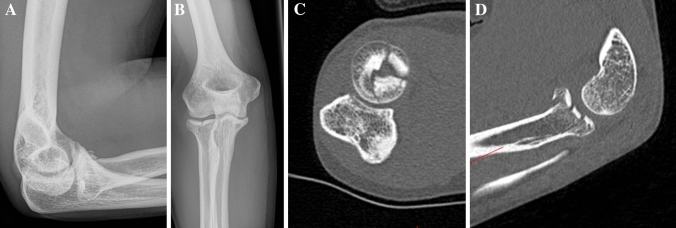
**a**, **b** Preoperative radiographs (lateral and anteroposterior) of a Mason type III fracture (Case1). **c** CT-scan in axial view and **d** in sagittal view showing the mutifragmental radial head

**Fig. 2:**
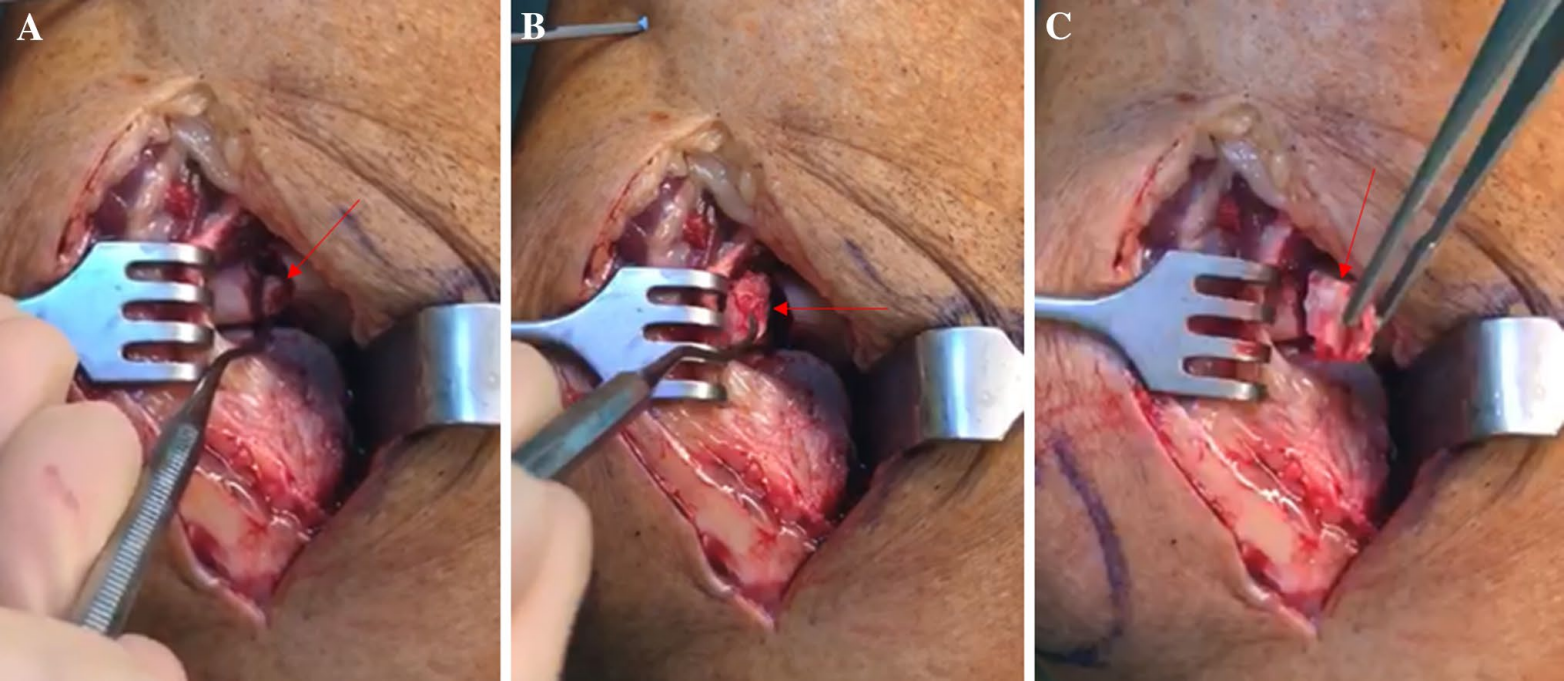
**a** Mobilization of an interposing cartilaginous fragment (red arrow) in by a dental hook (Case 1). **b** Intraoperative situs with visible interposing cartilaginous fragment. **c** Subsequent complete removal of the fragment

**Fig. 3:**
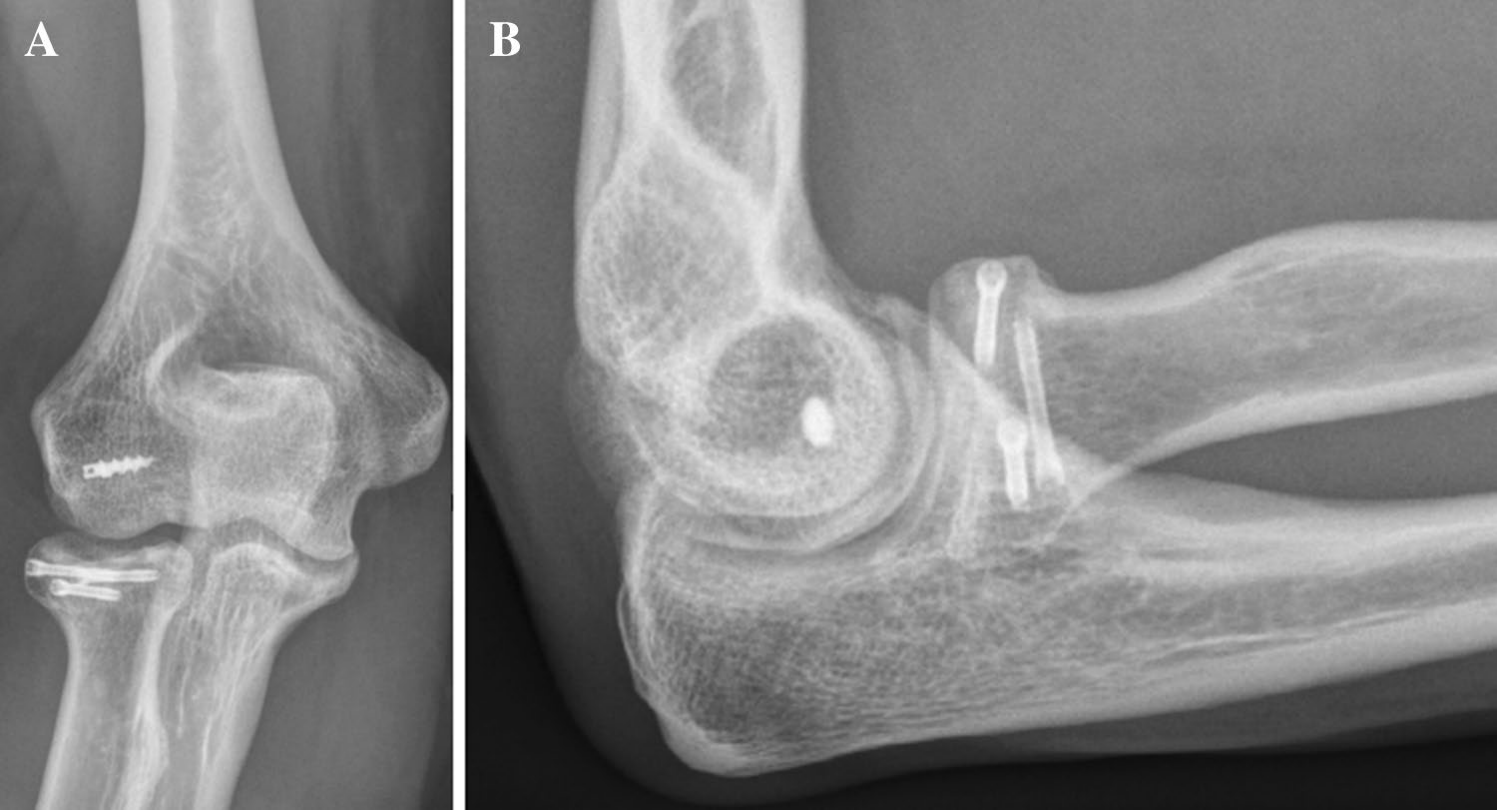
**a**, **b** Postoperative radiographs (anteroposterior and lateral) after radial head fixation with three CCS and reattached LCLC after 6 months (Case 1)

A 44-year-old female fell off her bicycle onto her outstretched right arm. A Mason type III fracture was revealed in the radiographs (Fig. 1). She had elbow motion from 70° to 90° with no possible supination or pronation. An additional CT-scan was performed, and the patient prepared for surgery. Surgery was performed 3 days after the initial accident using a Kaplan-approach to the lateral elbow. Intra-operatively, 2/3 of the LCLC (lateral collateral ligament complex) were found to be torn from its humeral origin resulting in a moderate varus instability under stress tests. There was no sign of medial instability. A cartilaginous lesion of the posterior capitellum (0.5 × 0.5 cm) was detected and the corresponding chondral flake interposed inside the fragmentally broken radial head (Fig. 2). Reduction was only possible by excising the flake and careful debridement of the joint. Osteosynthesis was then performed by three cannulated headless cannulated compressions screws (CCS) and the LCLC was reattached to its humeral origin using one anchor. Six months later, elbow motion was from 0° to 130°, pronation and supination each 90°, she reported no pain and radiographs showed a healed fracture (Fig. 3).

### Case 2

**Fig. 4:**
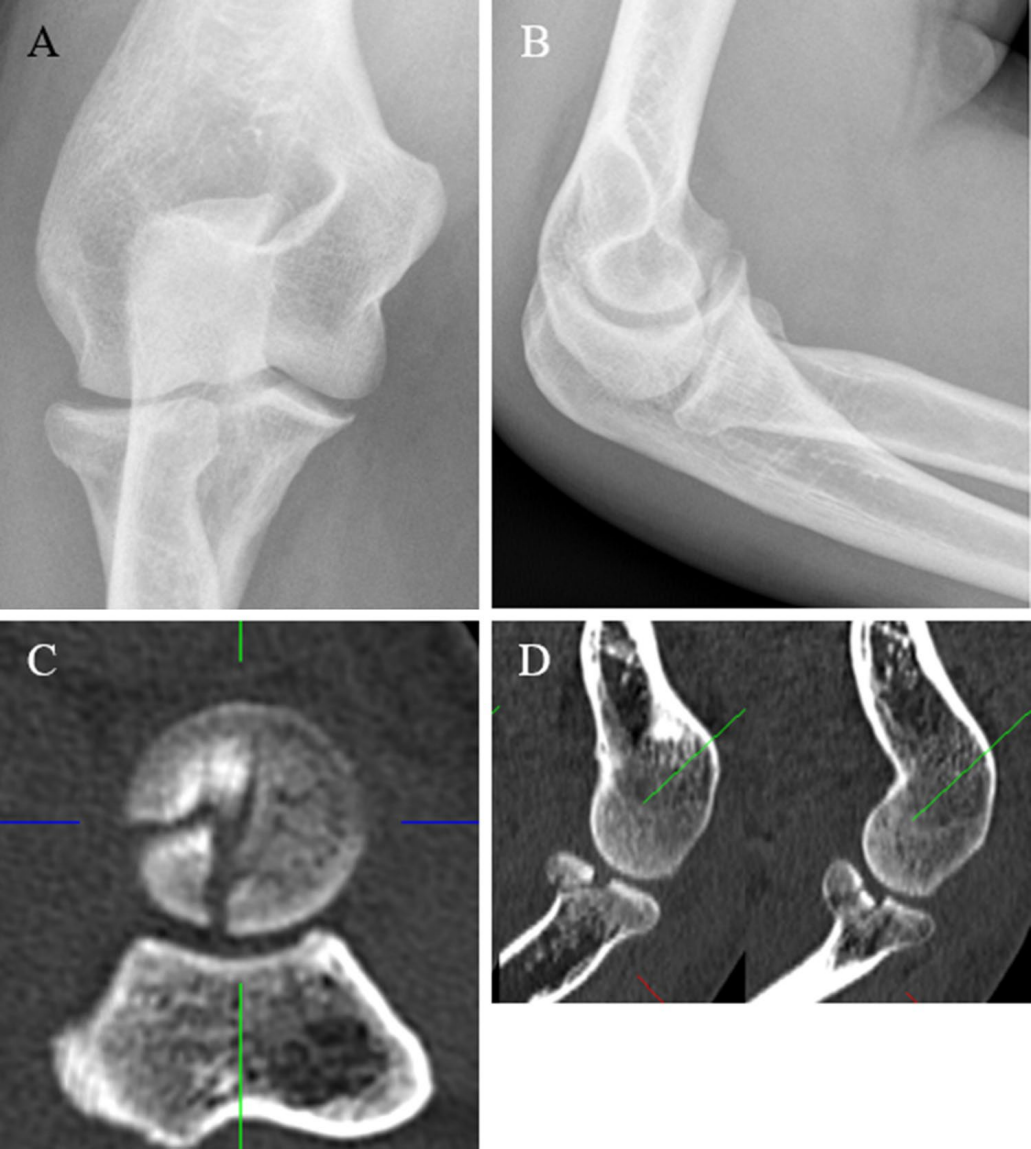
**a**, **b** Preoperative radiographs (anteroposterior and lateral) of a Mason type III fracture (Case 2). **c** CT-scan in axial view and **d** in sagittal view

**Fig. 5:**
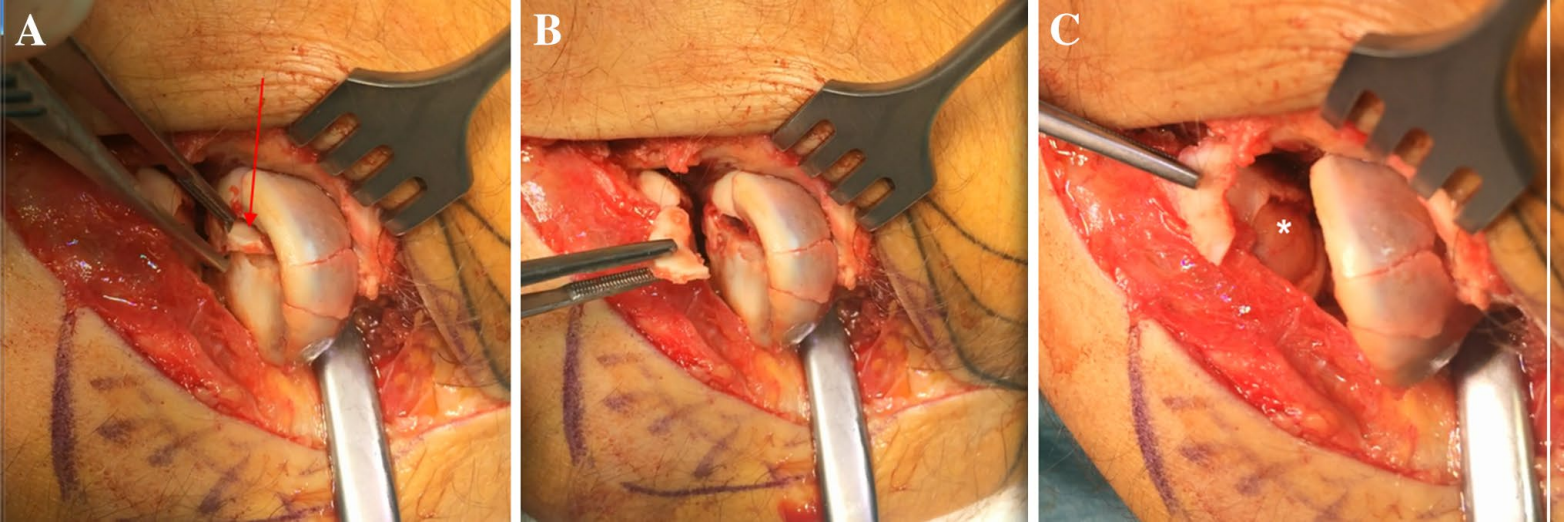
**a** Intraoperative situs (Case 2) with interposing cartilaginous fragment (red arrow). **b** Fragment is excised. **c** View of the capitellum with defect zone (indicated by asterisk)

**Fig. 6:**
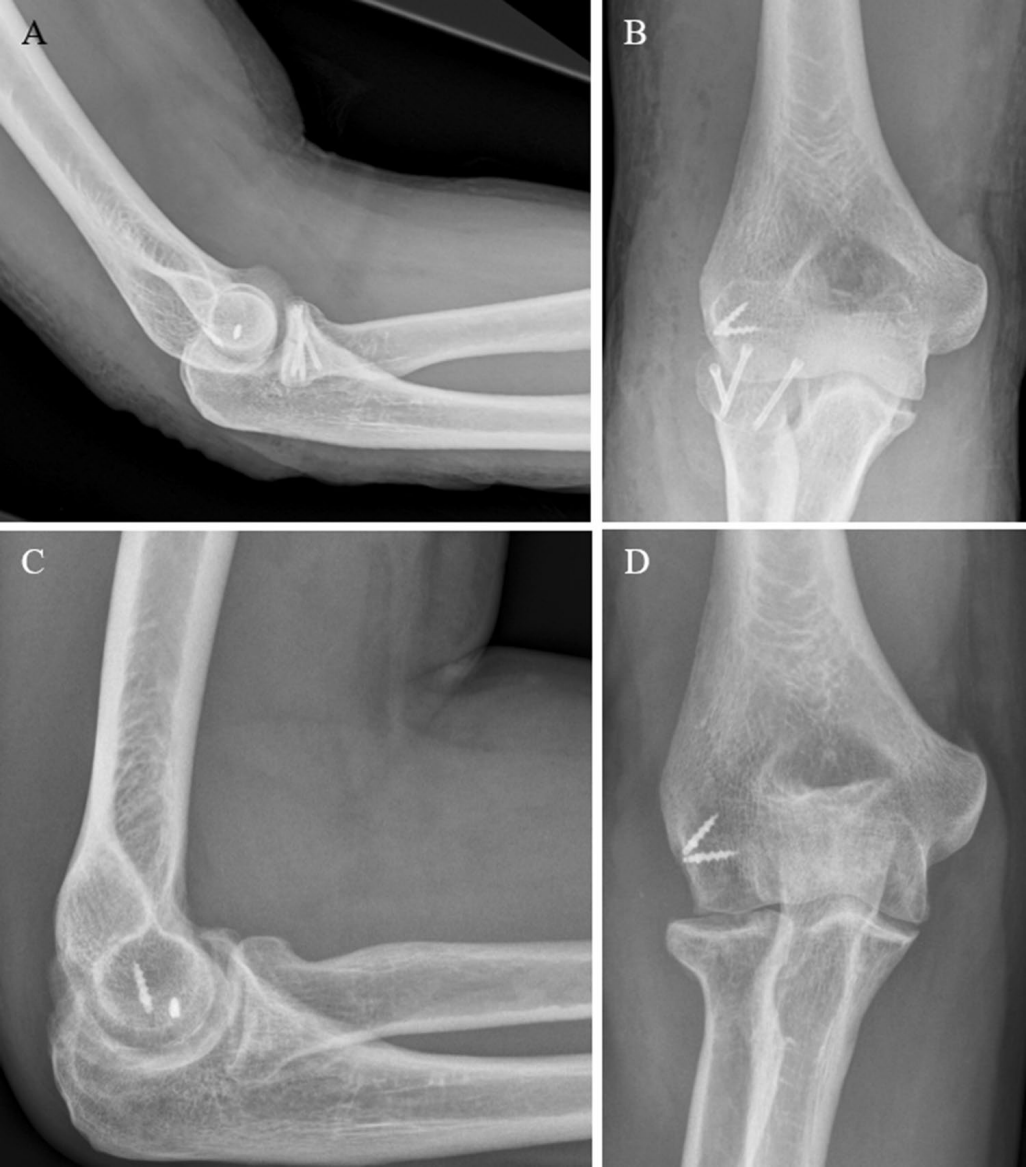
**a**, **b** Postoperative radiographs (lateral and anteroposterior) after radial head fixation with three CCS and reattached LCLC (Case 2). **c** and **d** after 6 months and metal removal

A 49-year-old male fell off a scaffold onto his outstretched right arm. The arm could not be moved due to severe pain. Radiographs and a CT-Scan was performed which revealed a Mason Type III fracture (Fig. 4). Surgery was performed 7 days later using a Kocher-approach [15]. There were no signs of ligamentous instability under stress tests. The LCLC was intact. A central cartilaginous flake, originating from the central aspect of the capitellum and interposed between the fracture fragments (Fig. 5) was excised and the fracture subsequently fixed by three CCS, as well as the LCLC reattached by two anchors (Fig. 6). The last follow-up was 26 months later. Elbow motion was 0° to 140°, pronation and supination being 90° each. There was no sign of pain and the radiographs showed a healed fracture.

### Case 3

**Fig. 7:**
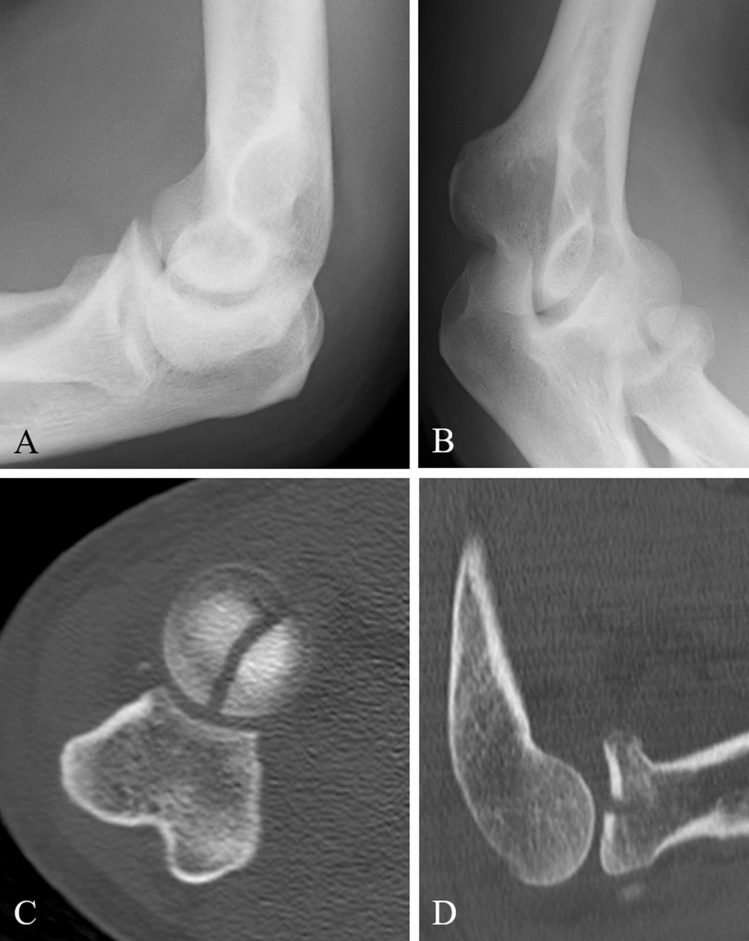
**a**, **b** Preoperative radiographs (lateral and oblique) of a Mason type II fracture (Case 3). **c** CT-scan in axial view and **d** in sagittal view

**Fig. 8:**
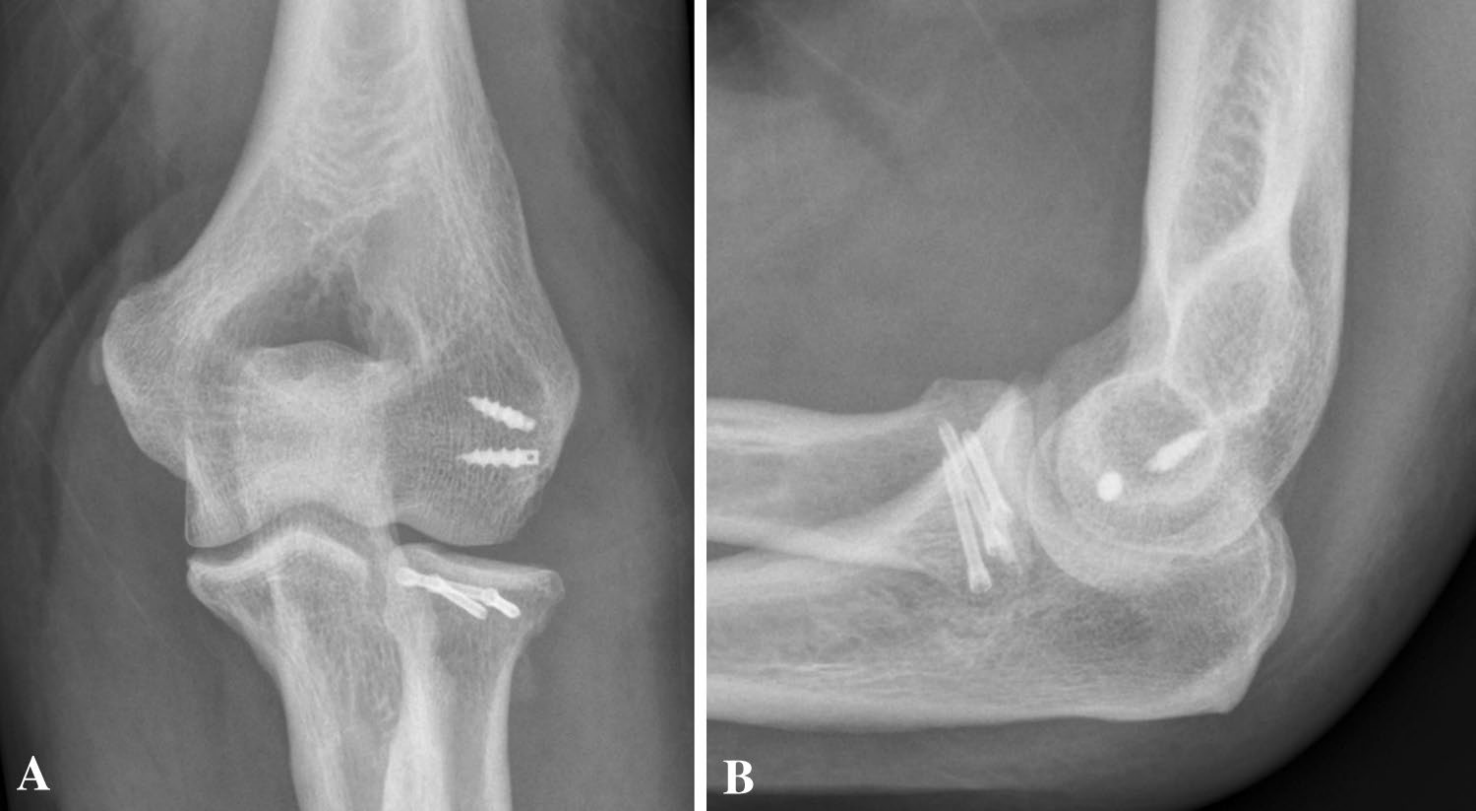
**a**, **b** Postoperative radiographs (lateral and anteroposterior) after radial head fixation with three CCS and reattached LCLC after 2 months (Case 3)

A 69-year-old male fell off his bicycle onto the outstretched left arm. The arm was immobilized by cast and the patient presented to us 8 days after the initial accident. Elbow motion was blocked in pronation and supination as well as painful. Radiographs and a CT-Scan, which revealed a Mason type II fracture, were obtained and surgery performed 2 days later (Fig. 7). The procedure consisted of a primary arthroscopy which was converted into an open Kaplan-approach upon fracture sight. The LCLC was found to be avulsed ½ of its humeral origin. Stress tests were positive for lateral but not for medial instability. A 0.5 × 1 cm measuring cartilaginous flake of the posterior aspect of the capitellum interposed in the fracture zone of the radial head. This flake was excised, and fixation performed using three CCS. The LCLC was reattached by two anchors (Fig. 8). Two months later, elbow motion was from 10° to 130°, pronation and supination 80° each. The patient was free of pain and radiographs revealed a healed fracture.

### Case 4

**Fig. 9:**
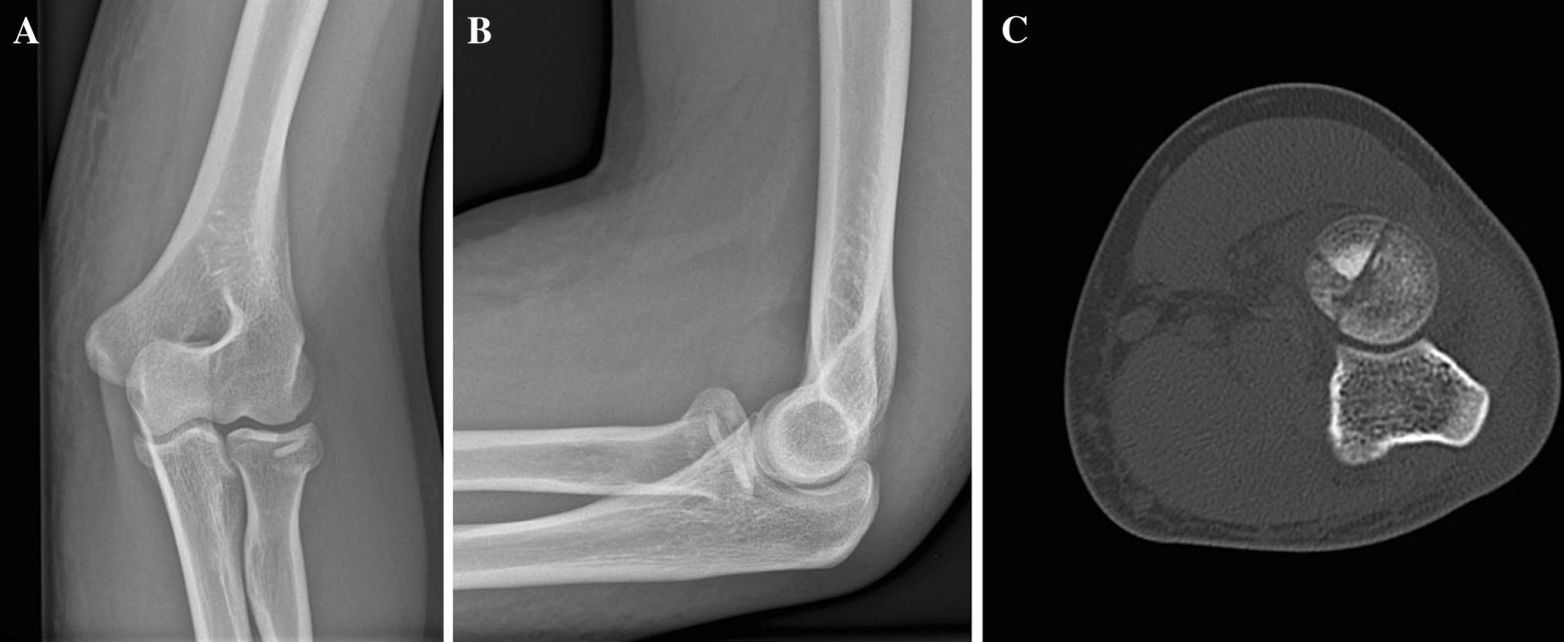
**a**, **b** Preoperative radiographs (anteroposterior and lateral) of a Mason type III fracture (Case 4). **c** CT-scan in axial view

**Fig. 10:**
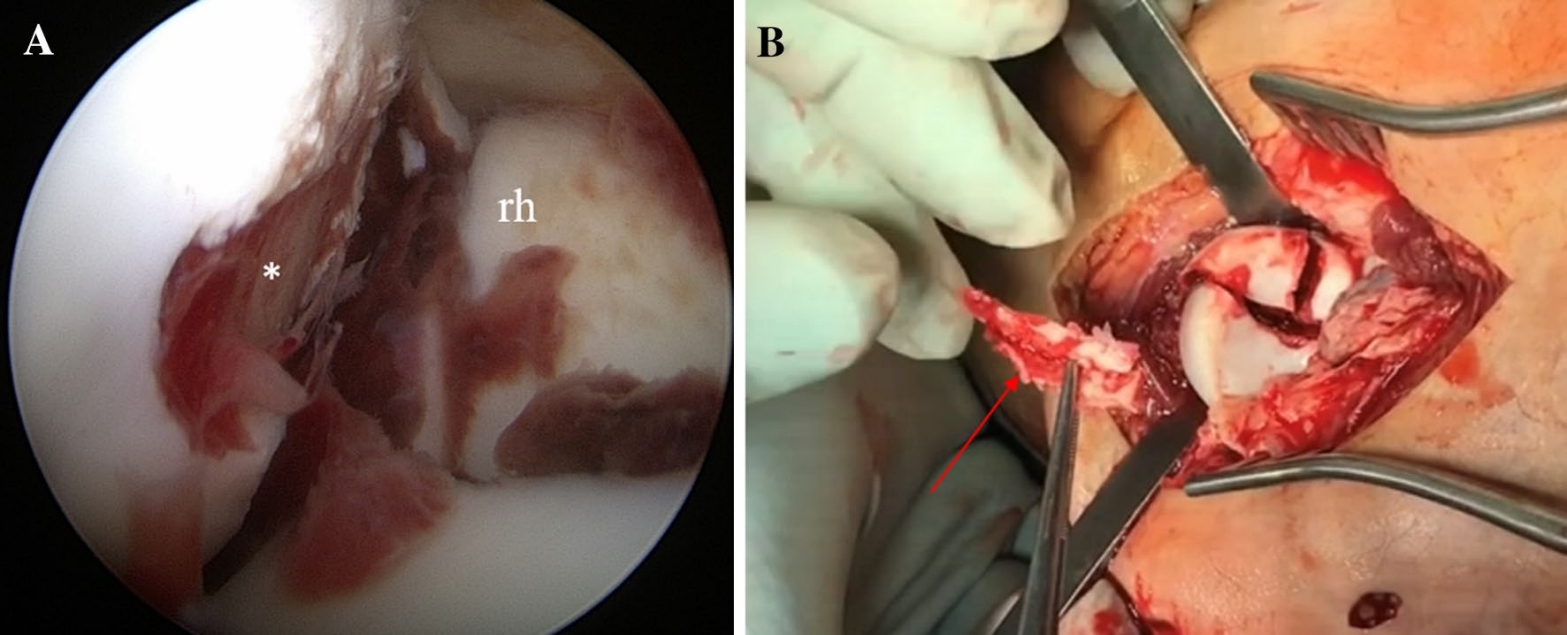
**a** Arthroscopic view of the capitellar cartilage defect (indicated by asterisk, *rh *radial head) (Case 4). **b** Subsequent removal of the interposing cartilaginous fragment (red arrow)

A 34-year-old-female fell off her bicycle on the way home from work. The left elbow was dislocated and reduced by herself. The first hospital to treat the patient recommended a conservative treatment with cast for 4 weeks. In rehabilitation after 8 weeks, she noticed increasing pain and crepitation. A CT-Scan was obtained, and at presentation elbow motion was 30–90° with full supination but no possible pronation. We detected an unhealed Mason type III fracture (Fig. 9). The patient was scheduled for surgery by primary arthroscopy, which was converted into an open Kocher-approach. The LCLC was partially avulsed and only half of it remained attached to the humerus in its original form. Stress tests indicated a moderate varus instability with intact medial instability. A cartilaginous flake interposed the radial fracture sight and was excised. The flake originated from the posterior capitellum (Fig. 10). After careful debridement of the radial head, the osteosynthesis was performed using four CCS and the LCLC was reattached by two anchors. After 3 months, elbow motion was from 15° to 120°, supination 90°, pronation 70°. There was some irritation around the medial epicondyle, but the ulnar nerve was not affected. Pain level was low overall (VAS = 2). Radiographs were obtained. The fracture consolidated.

There were no preoperative signs of such interposing capitellar fragments in any of the radiographic material we obtained. A CT-scan was performed in each case. The flakes were only detected and finally excised upon surgery. No reasonable refixation of these fragments to the capitellum could be performed in the presented cases.

## Discussion

This study presents the surprising cause of a primarily not possible reduction in three cases of a multifragmentary radial head fracture and one case of not possible conservative healing, caused by an interposing cartilage fragment originating from the capitellum.

Radial head fractures are among the most common bony injuries in adults. Most of them are isolated fractures but there are several typical concomitant injuries described in higher-energy traumas such as ligamentous and additional bony injuries [13]. These include the rupture of the medial or lateral collateral ligament (usually in cases of elbow (sub-)luxation), the interosseous membrane of the forearm (Essex-Lopresti) and or bony injuries such as a coronoid process fracture (terrible triad injury, together with an elbow dislocation) [16–21]. Injuries of the capitellum in pure radial head fractures, however, are rather rare. The participation of a merely cartilaginous fragment, which interposes in the radial fracture sight, is even rarer and has only been reported sparingly [21].

First descriptions of cases date back to 1916. Hitzrot described a case, where a capitellum fragment was trapped in the fracture line of a radial head fracture without being visible in radiographs [22]. Several identical cases have been reported in the following years [3, 4, 6, 7, 11, 23]. Caputo et al. introduced ten cases of trapped fragments in 2006. Plain radiographs did not reveal the full aspect of injury. In two cases, a preoperative CT-scan was performed which revealed a seemingly unharmed capitellum. All fragments were excised. Elbow function, forearm rotation and grip strength recovered completely in each case once the fragment was removed. There was no evidence of a higher arthrosis rate, compared to a sole radial head fracture in the mean follow-up of 11 months. The mechanism of injury in all cases was a fall on the outstretched arm [21].

The exact mechanism leading to a shear fracture of a cartilage fragment of the capitellum combined with a radial head fracture remains unknown. 60% of the axial load at the elbow joint is transmitted through the radiocapitellar surface [9, 24]. Some researchers believe that a certain elbow angle at the moment of impact of the radial head against the capitellum is responsible for cartilaginous shearing of the latter [4, 7, 25]. In this respect, we assume a short-term posterolateral subluxation of the radial head [21]. When the radial head subsequently repositions spontaneously, this fragment may become stuck in the fracture gap of the radial head. Furthermore, we assume that in most cases, this fragment rather tends to slip into the cubital joint and remains there unnoticed.

However, if it does get stuck in the fracture gap, even a Mason type I radial head fracture will probably not heal conservatively, as this fragment represents a morphological obstacle. This phenomenon was presented in Case 4. Removal of the fragment allowed stable reduction and fixation and caused the fracture to heal. A similar case was presented by Caputo et al. where a symptomatic nonunion could be successfully treated by fragment removal [21].

The natural course of conservative therapy of a Mason Type I radial head fracture with a blocking cartilage fragment of the capitellum is not known. However, it can be assumed that this fragment might play a role in some cases of symptomatic malunion or nonunion [6, 21, 26, 27].

In addition, there is no consensus yet on how to deal with merely cartilaginous capitellar fragments in radial head fractures. So far, fragment excision is recommended for fragments less than approximately 25–33% of the capitellar surface area [21]. Possible refixation tools are fibrin-sealant and bioabsorbable screws, pins or darts [28–31]. Most of these techniques have been described in knee surgery or osteochondrosis dissecans cases of the elbow [32–34].

The average size of the cartilage fragment appears to decrease with increasing severity of the radial head fracture. Nalbantonglu et al. found in their clinical results an average larger shearing fragment of the capitellum in Mason type II fractures than in Mason type III fractures [9]. A possible explanation might be that an intact radial head can cause more damage to the capitellum and in high-energy trauma the energy discharges through the bone rather than the cartilage, resulting in more bony injuries of the capitellum in Mason Type III fractures and more extensive merely cartilage injuries in Mason Type II and I fractures [9].

A fundamental question we asked ourselves when evaluating the cases was whether our classification of fractures was still valid after surgery. The LCLC was avulsed in three out of four cases with corresponding lateral instability. Rupture of the LCLC can typically occur in cases of external rotational forces combined with a valgus moment under axial loading. If the rotation exceeds a certain degree, dislocation may occur and tear the MCL additionally [35]. We could, however, not conclusively prove fracture dislocation, which would be a formal upgrade to a type IV fracture, in any of the cases. A common problem in the assessment of an isolated radial head fracture is the complete detection of all accompanying injuries [14]. Therefore, prolonged pain and limited elbow range of motion in terms of a mechanical block are red flags. These physical findings alone are sufficient indication for further work-up (such as needle aspiration and lidocaine injection to assess mechanical block and/or CT/MRI evaluation), and can lead to an operative intervention with the possibility that intra-articular cartilaginous fragments are creating a mechanical block to motion or, as in this series, are interposed within the radial head fracture serving as an impediment both to fracture healing and to elbow motion.

The question that remains is whether not every patient with a mechanical block and pain should get an early MRI. MR imaging is the most valuable technique to detect cartilaginous defects in a joint [9, 21]. Itamura et al. investigated concomitant injuries of radial head fractures Mason type II and III by MR imaging. They found in 29% capitellar osteochondral defects, in 96% capitellar bone bruise and in 92% of 24 cases loose bodies [10]. Therefore, it should be discussed whether MRI should be performed in every case of a non-properly healing radial head fracture that presents with prolonged pain and mechanical block to detect concomitant injuries [14].

Of course, there are some limitations associated with this study. One is the small number of cases that could be included in this study. Nalbantoglu et al. reported a capitellar cartilage defect in 10 of 51 cases. Overall, we assume a high number of undetected cases.

Additionally, the follow-up periods are very heterogeneous, which means that an exact statement about long-term function of the presented cases is only possible to a limited extent.

In summary, this case series demonstrates rare cases of interposing capitellar cartilage fragments in radial head fractures, which cannot be seen on radiological images (plain radiographs and CT) and may therefore act as an unusual sight during surgery. High suspicion is indicated in cases of mechanical irritation and pain after conservatively treated radial head fractures, as an interposing capitellar cartilage fragment may be the cause of non-healing, mechanical block, or pain and should be treated favorably by open surgery or arthroscopy with debridement and removal of any loose cartilaginous fragments.

## References

[1] Kaas L, van Riet RP, Vroemen JPAM, Eygendaal D (2010). The epidemiology of radial head fractures. J Shoulder Elb Surg.

[2] Duckworth AD, Clement ND, Jenkins PJ, Aitken SA, Court-Brown CM, McQueen MM (2012). The epidemiology of radial head and neck fractures. J Hand Surg-Am.

[3] Geel CW, Palmer AK, Ruedi T, Leutenegger AF (1990). Internal fixation of proximal radial head fractures. J Orthop Trauma.

[4] Heim U, Trub HJ (1978). Experiences with primary osteosynthesis in radial head fractures. Helv Chir Acta.

[5] Mason ML (1954). Some observations on fractures of the head of the radius with a review of one hundred cases. Br J Surg.

[6] Poulsen JO, Tophoj K (1974). Fracture of the head and neck of the radius. Follow-up on 61 patients. Acta Orthop Scand.

[7] Ward WG, Nunley JA (1988). Concomitant fractures of the capitellum and radial head. J Orthop Trauma.

[8] van Riet RP, van den Bekerom M, Van Tongel A, Spross C, Barco R, Watts AC (2020). Radial head fractures. Shoulder Elb.

[9] Nalbantoglu U, Gereli A, Kocaoglu B, Aktas S, Turkmen M (2008). Capitellar cartilage injuries concomitant with radial head fractures. J Hand Surg Am.

[10] Itamura J, Roidis N, Mirzayan R, Vaishnav S, Learch T, Shean C (2005). Radial head fractures: MRI evaluation of associated injuries. J Shoulder Elb Surg.

[11] Milch H (1931). Unusual fractures of the capitulum humeri and the capitulum radii. J Bone Joint Surg Am.

[12] Johnston GW (1962). A follow-up of one hundred cases of fracture of the head of the radius with a review of the literature. Ulster Med J.

[13] van Riet RP, Morrey BF, O'Driscoll SW, Van Glabbeek F (2005). Associated injuries complicating radial head fractures: a demographic study. Clin Orthop Relat Res.

[14] Burkhart KJ, Wegmann K, Muller LP, Gohlke FE (2015). Fractures of the radial head. Hand Clin.

[15] Petersen KA, Siesel C, Miller ET (2019). Radial head replacement through a Kocher approach. J Orthop Trauma.

[16] Arner O, Ekengren K, Von Schreeb T (1957). Fractures of the head and neck of the radius; a clinical and roentgenographic study of 310 cases. Acta Chir Scand.

[17] Davidson PA, Moseley JB, Tullos HS (1993). Radial head fracture. A potentially complex injury. Clin Orthop Relat Res.

[18] Essex-Lopresti P (1951). Fractures of the radial head with distal radio-ulnar dislocation; report of two cases. J Bone Joint Surg Br.

[19] Hotchkiss RN (1997). Displaced fractures of the radial head: internal fixation or excision?. J Am Acad Orthop Surg.

[20] Liu SH, Henry MH (1995). Fracture of the radial head with ulnar collateral ligament rupture. J Shoulder Elb Surg.

[21] Caputo AE, Burton KJ, Cohen MS, King GJ (2006). Articular cartilage injuries of the capitellum interposed in radial head fractures: a report of ten cases. J Shoulder Elb Surg.

[22] Hitzrot J (1916). Comment on open reduction of the capitellum. Ann Surg.

[23] Newman JH (1983). Radius fractures and damage to the capitulum humeri. Injury.

[24] Morrey BF, An KN, Stormont TJ (1988). Force transmission through the radial head. J Bone Joint Surg Am.

[25] Palmer I (1961). The validity of the rule of alternativity in traumatology. Acta Chir Scand.

[26] Bakalim G (1970). Fractures of radial head and their treatment. Acta Orthopaedica Scandinavica.

[27] Radin EL, Riseborough EJ (1966). Fractures of the radial head. A review of eighty-eight cases and analysis of the indications for excision of the radial head and non-operative treatment. J Bone Joint Surg Am.

[28] Maletius W, Lundberg M (1994). Refixation of large chondral fragments on the weight-bearing area of the knee joint: a report of two cases. Arthroscopy.

[29] Morris JK, Weber AE, Morris MS (2016). Adolescent femoral chondral fragment fixation with poly-l-lactic acid chondral darts. Orthopedics.

[30] Sano S, Rokkaku T, Saito S, Tokunaga S, Abe Y, Moriya H (2005). Herbert screw fixation of capitellar fractures. J Shoulder Elb Surg.

[31] Uchida S, Utsunomiya H, Taketa T, Sakoda S, Hatakeyama A, Nakamura T, Sakai A (2015). Arthroscopic fragment Fixation using hydroxyapatite/poly-l-lactate acid thread pins for treating elbow osteochondritis dissecans. Am J Sport Med.

[32] Inoue G (1991). Bilateral osteochondritis dissecans of the elbow treated by Herbert screw fixation. Br J Sports Med.

[33] de Graaff F, Krijnen MR, Poolman RW, Willems WJ (2011). Arthroscopic surgery in athletes with osteochondritis dissecans of the elbow. Arthroscopy.

[34] Harada M, Ogino T, Takahara M, Ishigaki D, Kashiwa H, Kanauchi Y (2002). Fragment fixation with a bone graft and dynamic staples for osteochondritis dissecans of the humeral capitellum. J Shoulder Elb Surg.

[35] Kaas L, Turkenburg JL, van Riet RP, Vroemen JP, Eygendaal D (2010). Magnetic resonance imaging findings in 46 elbows with a radial head fracture. Acta Orthop.

